# A Review of Thyroid Rests: From Embryology to Clinical Management

**DOI:** 10.7759/cureus.95027

**Published:** 2025-10-21

**Authors:** Ghezlan Aldawas, Sherif Ragab

**Affiliations:** 1 Department of Otolaryngology, Head and Neck Surgery, Farwaniya Hospital, Kuwait City, KWT; 2 Department of Otolaryngology, Head and Neck Surgery, Ministry of Health, Kuwait, Kuwait City, KWT

**Keywords:** ectopic thyroid, embryology, misdiagnosis, multidisciplinary management, neck masses, thyroid rests

## Abstract

Ectopic thyroid tissue (ETT) is a rare developmental anomaly often misidentified as other pathologies like parathyroid adenomas or metastatic lymphadenopathy. This can lead to unnecessary surgical interventions and diagnostic delays. This narrative review aims to synthesize current knowledge on the embryological origins, anatomical distribution, diagnostic challenges, and management strategies of ETT. A comprehensive literature search was conducted by searching PubMed, Google Scholar, and Scopus databases, covering publications from January 2015 to January 2025. Fifteen peer-reviewed publications, including case reports, case series, reviews, and original research, were selected for inclusion. Ectopic thyroid tissue (ETT) most commonly manifests as a lingual thyroid, situated at the base of the tongue. Diagnosis is challenging due to overlapping imaging characteristics with neoplastic or hyperfunctioning parathyroid tissue. A multimodal approach is essential, combining imaging (ultrasound, CT, MRI) with functional assessments (scintigraphy) and histological confirmation (FNA). Management is individualized, with observation for asymptomatic cases and surgical intervention for symptomatic or suspicious lesions. A multidisciplinary team (MDT) approach is critical for accurate diagnosis and to prevent unnecessary procedures. This review underscores the importance of structured diagnostic protocols to improve outcomes for patients with ETT.

## Introduction and background

Ectopic thyroid tissue (ETT), although rare, holds significant clinical importance due to its potential misidentification as parathyroid adenomas or metastatic lymphadenopathy, often resulting in unwarranted surgical interventions [1]. ETT refers to the presence of thyroid tissue located outside its normal anatomical position in the anterior neck. These aberrant deposits arise from disruptions in the embryological migration of the thyroid gland and may be found in various anatomical regions, including the tongue base (foramen cecum), mediastinum, and lateral neck compartments [2,3]. Recent evidence indicates variable prevalence rates across populations, with the lingual thyroid remaining the most frequent presentation [4]. ETT is frequently asymptomatic but can mimic neoplastic conditions such as parathyroid adenomas, lymphadenopathy, or metastatic lesions, leading to diagnostic confusion and unnecessary procedures [1]. With the increasing use of high-resolution imaging modalities such as ultrasonography and computed tomography, incidental detection of ETT has become more common during evaluations for unrelated conditions [5]. Diagnostic confirmation typically involves multimodal assessment, incorporating imaging, scintigraphy, and histopathological evaluation to ensure accurate differentiation from other cervical pathologies. Therefore, a thorough understanding of thyroid embryology, anatomical variants, and pathological behavior is essential for clinicians [2,3]. This review aims to elucidate the embryological origins, anatomical distribution, diagnostic challenges, and management strategies of ETT, emphasizing the importance of multidisciplinary, evidence-based approaches to improve recognition and reduce overtreatment.

## Review

Methdos

This study is a narrative review. A comprehensive literature search was conducted using PubMed, Google Scholar, and Scopus databases, covering publications from January 2010 to January 2025. Search terms included “ectopic thyroid,” “thyroid rests,” “thyroid embryology,” and “misdiagnosis of ectopic thyroid.” Only English-language articles were considered, including case reports, case series, reviews, and original research. A total of 19 peer-reviewed publications were selected for inclusion.

Embryology of thyroid rests

The thyroid gland originates from both median and lateral embryological components, forming a complex endocrine structure. It begins as a median endodermal thickening at the foramen cecum in the primitive pharynx and descends anteriorly along the pharyngeal gut as a bilobed diverticulum, forming the thyroglossal duct during its migration to the anterior neck, where it ultimately resides anterior to the trachea [2,6]. Disruptions in this descent can result in ectopic thyroid tissue along the migration pathway, ranging from the tongue base (foramen cecum, lingual thyroid) to the mediastinum. Rico and Lung described dual embryological origins of the thyroid: the median endodermal anlage and lateral paired anlagen derived from the fourth pharyngeal pouch. While the median component contributes the majority of thyroid tissue, aberrations in fusion or migration of the lateral elements may lead to ectopic remnants [2]. These rests may remain clinically silent or present with mass effects depending on their size and location.

Additional developmental anomalies can explain ectopic thyroid tissue outside the midline thyroglossal tract. Gupta et al. noted that failure of thyroglossal duct involution may result in epithelial-lined cystic or solid thyroid tissue, commonly manifesting as thyroglossal duct cysts near the hyoid bone [3]. These midline lesions are the most frequent form of ETT in pediatric and young adult populations. In contrast, intratracheal thyroid rests arise from abnormal adhesions between the thyroid anlage and the developing tracheal wall. Pantha et al. reported that such aberrant connections can lead to exotracheal proliferations, potentially obstructing the airway [7]. Zhang et al. corroborated this mechanism, documenting pediatric cases of tracheal obstruction due to intraluminal thyroid rests misdiagnosed as tumors or papillomas [8]. Understanding these embryological variations is critical not only for accurate diagnosis but also for surgical planning, as inadvertent removal or retention of ectopic tissue may impact thyroid function and complicate treatment.

Common and rare anatomical locations

Ectopic thyroid tissue exhibits considerable anatomical variability, with the most frequent sites aligning with the thyroglossal duct pathway, consistent with its embryological origin. Noussios et al. identified the lingual thyroid, located at the base of the tongue, as the most prevalent form, accounting for approximately 90% of all ETT cases [6]. Other commonly reported sites include sublingual and suprahyoid regions (Table 1). These locations may become symptomatic due to mass effect, particularly during periods of increased physiological demand such as puberty or pregnancy. Accurate localization is essential to avoid unnecessary surgical excision, especially in cases where the ectopic focus represents the sole functioning thyroid tissue. Imaging and functional assessments are critical for differentiating lingual and sublingual rests, which may present with dysphagia, dysphonia, or airway obstruction.

**Table 1:** Anatomic distribution of ectopic thyroid tissue and typical diagnostic mimics Recognizing these patterns aids in targeted evaluation and reduces unnecessary surgery. Refrences [6,9–11].

Location	Frequency	Common Mimics
Lingual thyroid	Common	Hypertrophic lingual tonsil, vallecular cyst
Subhyoid/Suprahyoid	Common	Thyroglossal duct cyst, midline dermoid cyst
Mediastinal	Rare	Thymoma, lymphadenopathy, metastatic carcinoma
Lateral neck	Rare	Metastatic lymph node, branchial cleft cyst
Intratracheal	Rare	Tracheal tumor, granuloma
Sublingual	Uncommon	Ranula, salivary gland tumor
Intra-abdominal/Liver	Very rare	Hepatic adenoma, metastasis

Ectopic thyroid tissue in atypical, non-midline locations poses additional diagnostic and therapeutic challenges. Cvasciuc et al. proposed a classification for retrosternal goiters based on their mediastinal position and proximity to vital thoracic structures, noting that mediastinal ETT is often misinterpreted as intrathoracic masses [9]. Di Stefano et al. described a rare case of hepatic ectopic thyroid tissue, incidentally discovered during liver imaging, underscoring the need to consider ETT beyond the cervical and thoracic regions [10]. Similarly, Sugiyama et al. reported a lateral cervical ectopic thyroid cyst initially misdiagnosed as a second branchial cleft cyst, illustrating the diagnostic complexity of lateral neck lesions in the absence of embryological context [11]. These cases highlight the importance of detailed anatomical and embryological knowledge in differential diagnosis. Given that ETT may arise outside the expected midline tract, its imaging characteristics can overlap with nodal disease and parathyroid lesions, increasing the risk of misdiagnosis.

Common misdiagnoses

Ectopic thyroid tissue (ETT) is frequently misinterpreted as other cervical or mediastinal pathologies, including parathyroid adenomas and metastatic lymphadenopathy, due to its atypical anatomical presentation. The imaging characteristics of ETT often overlap with those of neoplastic or hyperfunctioning parathyroid tissue, complicating accurate diagnosis. Raji et al. described ETT as a “great mimicker,” noting that its radiographic and sonographic features can closely resemble those of thyroid carcinomas and adenomas, potentially leading to diagnostic errors during initial evaluation [1]. These cases underscore the importance of including ectopic thyroid and parathyroid tissue in the differential diagnosis of atypical neck masses to avoid unnecessary interventions and treatment delays. Such diagnostic pitfalls reinforce the need for a multimodal diagnostic strategy that integrates anatomical and functional imaging.

Diagnostic approach

The accurate diagnosis of ETT requires a comprehensive, multimodal approach that combines imaging, cytological evaluation, and histopathological confirmation. Ultrasound (US) remains the primary imaging modality, particularly in pediatric and outpatient settings, due to its accessibility and ability to assess echotexture, vascularity, and anatomical positioning. Tritou et al. emphasized the utility of a structured ultrasonographic checklist for pediatric thyroid evaluation, which enhances the detection and characterization of ectopic foci by focusing on imaging patterns and anatomical relationships [5]. However, US alone may be insufficient to reliably distinguish ETT from other pathologies such as lymphadenopathy or parathyroid lesions, necessitating further investigation.

For deeper or atypically located lesions, advanced imaging techniques such as computed tomography (CT), magnetic resonance imaging (MRI), and nuclear medicine studies are often required. Elsayeed et al. demonstrated the value of thyroid scintigraphy in confirming the functional nature of ectopic thyroid tissue, particularly in the absence of orthotopic thyroid and in the presence of elevated thyroid-stimulating hormone (TSH) levels [12]. Zhou and Chen further highlighted the utility of single-photon emission computed tomography (SPECT)/CT in localizing challenging mediastinal parathyroid adenomas [13]. Recent studies have underscored the pivotal role of hybrid imaging modalities, including SPECT/CT and PET/CT, in enhancing diagnostic precision, anatomical localization, and overall management of ectopic thyroid lesions. [14]. When imaging results are inconclusive, fine-needle aspiration (FNA) cytology becomes essential. Salman et al. discussed the diagnostic role of histological markers and hormone profiling in differentiating ETT from neoplastic processes [15]. Once ETT is accurately localized and characterized, management decisions are guided by clinical symptoms, functional status, and associated risk factors.

Management and surgical decision-making

The management of ETT is highly individualized and depends on several factors, including symptomatology, anatomical location, hormonal activity, and the potential for malignancy. In asymptomatic patients with benign imaging features, conservative management is generally preferred. Bilezikian’s guidelines advocate for observation in cases where malignancy or parathyroid disease is not evident and clinical risk is minimal, thereby avoiding unnecessary surgical intervention [16]. Regular monitoring through imaging and endocrine evaluation allows clinicians to detect changes in size, function, or symptomatology, thereby minimizing the risk of overtreatment.

Surgical intervention is indicated in cases of compressive symptoms, recurrent infections, cosmetic concerns, or hormonal dysregulation. However, surgical decision-making should not rely solely on clinical presentation; embryological origin and anatomical relationships must also be considered. Rico and Lung emphasized that the final anatomical position of thyroid remnants is determined by developmental pathways, which influence the complexity of surgical excision [2]. Special care must be taken to avoid injury to adjacent structures such as the recurrent laryngeal nerve and parathyroid glands.

In cases involving intratracheal, mediastinal, or retrosternal ETT, surgical access is more complex and often necessitates collaboration with thoracic surgeons. Pantha et al. reported favorable outcomes following multidisciplinary surgical management of intratracheal ETT involving both ENT and thoracic specialists [7]. Similarly, Zhang et al. described the use of both endoscopic and open surgical techniques tailored to the size and location of the ectopic tissue, demonstrating the feasibility of minimally invasive approaches when appropriate [8]. These examples underscore the importance of preoperative planning, detailed imaging, and multidisciplinary coordination, particularly in anatomically challenging cases (Table 2). Optimal outcomes are achieved by balancing surgical risk, anatomical accessibility, and patient-specific considerations.

**Table 2:** Case report summary of uncommon locations, misdiagnosis, diagnostic tools and suggested management US = ultrasound; CT = computed tomography. References  [8,10,17,18].

Author(s)	Location	Misdiagnosis	Diagnostic Tool	Management
Connolly et al. [17]	Piriform sinus	Thyroid mass	CT, Histopathology	Surgical excision
Di Stefano et al. [10]	Liver	Hepatic tumor	CT, Histopathology	Observation
Roztoczyńska et al. [18]	Cervical	Thyroid hyperplasia	US, Histopathology	Surgery
Zhang et al. [8]	Trachea	Tracheal tumor	CT, Endoscopy	Surgical excision

**Figure 1:**
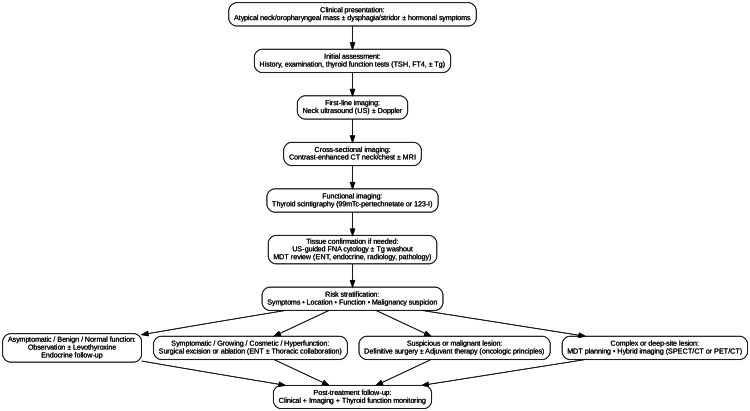
Diagnostic and management flowchart for ectopic thyroid tissue (ETT) US = ultrasound; CT = computed tomography; MRI = magnetic resonance imaging; FNA = fine-needle aspiration; MDT = multidisciplinary team; Tg = thyroglobulin. Created by the authors based on current evidence [5,7–9,12–16,19].

Importance of multidisciplinary evaluation

The evaluation and management of ETT, particularly in atypical anatomical locations, benefit significantly from a multidisciplinary team (MDT) approach. Collaboration among endocrinologists, radiologists, pathologists, and surgeons enhances diagnostic accuracy and informs appropriate treatment strategies. Hutsebaut et al. emphasized the value of preoperative consultations involving radiology and pathology teams, which facilitate shared decision-making and help avoid unnecessary surgical interventions in cases of benign ectopic tissue [19]. This is especially critical when imaging findings are ambiguous or when the presence of functional orthotopic thyroid tissue is uncertain. Early involvement of pathologists allows for contextual interpretation of FNA results, reducing the risk of overdiagnosis or misclassification.

Nuclear medicine also plays a pivotal role in surgical planning, particularly in identifying ectopic tissue that may be missed by conventional imaging. Zhou and Chen demonstrated the value of SPECT/CT in localizing ectopic lesions in the mediastinum, highlighting the role of functional imaging when conventional modalities are inconclusive [13]. Salman et al. advocated for a dual-modality approach that integrates anatomical imaging (e.g., US, CT) with physiological assessment (e.g., scintigraphy, SPECT) to improve diagnostic precision [15]. This integrated strategy supports more accurate lesion characterization and facilitates informed decision-making regarding observation, medical management, or surgical intervention. Coordinated multidisciplinary care is especially advantageous in complex or rare anatomical presentations, reducing the likelihood of both undertreatment and overtreatment.

Discussion

The findings of this review support a structured, stepwise diagnostic approach for ectopic thyroid tissue (ETT), beginning with anatomical imaging, followed by functional assessment in cases where orthotopic thyroid tissue is absent or indeterminate, and reserving histological confirmation for discordant findings. This protocol is best implemented within a multidisciplinary team (MDT) framework to ensure diagnostic accuracy and appropriate management.

ETT presents a diagnostic challenge due to its variable anatomical distribution and its potential to mimic other pathological entities. These anomalies arise from disruptions in the embryological descent of the thyroid gland, resulting in ectopic localization. While lingual and sublingual thyroid rests are most commonly reported, rare sites such as the liver, trachea, and mediastinum have also been documented. These atypical presentations may be misinterpreted as unrelated lesions, underscoring the need for a systematic diagnostic process [7,9-11].

Initial imaging modalities should include ultrasound (US), computed tomography (CT), and radionuclide scintigraphy, which together provide complementary insights into anatomical structure and functional status. In cases where imaging is inconclusive, histopathological evaluation is essential to guide therapeutic decisions [15]. Prior to surgical excision, clinicians must confirm the presence and function of orthotopic thyroid tissue, particularly in atypical locations, where scintigraphy or single-photon emission computed tomography (SPECT) may be warranted. Misdiagnosis can result in unnecessary or inappropriate surgical interventions, especially when ETT is mistaken for parathyroid adenomas or metastatic disease [13].

The literature supports a triad of diagnostic steps for atypical lesions: (1) targeted imaging using US, CT, and scintigraphy; (2) correlation with laboratory findings; and (3) histopathological confirmation prior to surgical decision-making. MDT involvement including radiology, nuclear medicine, pathology, and endocrine surgery enhances diagnostic precision and facilitates optimal treatment planning [19,14]. This collaborative approach reduces the risk of overtreatment and delays in care, as emphasized by Hutsebaut et al. [19] and Zhou and Chen [13]. Further standardization of imaging protocols and the development of radiologic checklists may improve diagnostic consistency and ensure timely, appropriate intervention for patients with suspected ETT.

Key points

Ectopic thyroid tissue (ETT) can closely resemble parathyroid or nodal disease, making functional and histological confirmation necessary before any surgical intervention. A multimodal diagnostic approach that combines anatomical and functional imaging such as ultrasound, CT, MRI, scintigraphy, or SPECT together with fine-needle aspiration cytology, allows for more accurate diagnosis. Involvement of a multidisciplinary team (MDT) reduces diagnostic delays and helps to avoid unnecessary surgery. Management should always be based on the patient’s symptoms and thyroid function, with careful observation being appropriate in many cases of incidentally detected ETT.

## Conclusions

Ectopic thyroid tissue represents a rare but clinically significant developmental anomaly that may be mistaken for more common or neoplastic conditions. These aberrant tissues, resulting from defective embryological migration, can appear in atypical cervical or mediastinal locations and mimic entities such as parathyroid adenomas or metastatic disease. A thorough understanding of thyroid embryogenesis and anatomical variability is essential for clinicians to avoid diagnostic oversights.

Effective management of ETT requires a cautious and methodical approach, integrating advanced imaging techniques, cytological evaluation, and multidisciplinary collaboration. Central to this process is maintaining a high index of clinical suspicion, which enables precise, patient- centered therapeutic planning while minimizing the risk of unnecessary interventions. This review underscores the importance of structured diagnostic protocols and coordinated care in improving outcomes for patients with ectopic thyroid tissue.
